# Research Domain Criteria in NIMH Grants Characterized Using Large Language Models

**DOI:** 10.1001/jamanetworkopen.2024.59371

**Published:** 2025-02-12

**Authors:** Roy H. Perlis

**Affiliations:** 1 Center for Quantitative Health, Massachusetts General Hospital, Boston; 2 Department of Psychiatry, Harvard Medical School, Boston, Massachusetts; 3 AI Editor, *JAMA Network Open*, Chicago, Illinois

## Abstract

**Importance:**

Over the past decade, the leadership of the National Institute of Mental Health (NIMH) has emphasized the importance of a transdiagnostic approach to psychiatric investigation using Research Domain Criteria (RDoC) mapping more closely to neurobiology.

**Objective:**

To investigate whether research support from the NIMH for individual RDoC domains and for transdiagnostic investigation has changed over time and has had differential impact in terms of publication, citations, or patent filings.

**Design, Setting, and Participants:**

In this longitudinal cohort study, all R01, R21, and R03 studies funded by the NIMH between January 2003 and December 2023 were identified via the National Institutes of Health RePORTER database. Their abstracts were characterized in terms of RDoC domains (negative valence, positive valence, cognition, social, arousal, and sensorimotor) using a large language model.

**Main Outcomes and Measures:**

Primary outcomes were publications, citation impact estimated at 5 years from the index year of funding, and patents, examined using regression models adjusted for other grant characteristics.

**Results:**

Among 8897 R01, R03, and R21 projects, reflecting $17.7 billion of investment, abstracts of 3141 (35.3%) reflected negative valence; 1344 (15.1%), positive valence; 2781 (31.3%), cognition; 1607 (18.1%), social; 343 (3.9%), arousal; and 571 (6.4%), sensorimotor domains. A total of 1793 (20.2%) incorporated a transdiagnostic perspective. Positive and social domains were associated with fewer publications (difference, −1.13 [95% CI, −2.11 to −0.15] and −2.23 [95% CI, −3.15 to −1.30], respectively) and lesser citation impact (difference, −0.47 [95% CI, −0.75 to −0.18] and −1.19 [95% CI, −1.46 to −0.91], respectively) at 5 years. Social (adjusted odds ratio [AOR], 0.11; 95% CI, 0.04-0.23) and cognitive (AOR, 0.66; 95% CI, 0.48-0.89) domains and transdiagnostic proposals (AOR, 0.37; 95% CI, 0.21-0.60) were associated with lower likelihood of patent filing.

**Conclusions and Relevance:**

In this study of NIMH funding, grants reflecting different RDoC domains differed substantially in their scientific impact in terms of publications, citations, and patent generation. The findings suggest that large language models represent a promising approach to characterizing research proposals at scale, which may be useful in guiding resource allocation to maximize scientific return on investment.

## Key Points

**Question:**

Has research support from the National Institute of Mental Health (NIMH) for individual Research Domain Criteria (RDoC) domains and for transdiagnostic investigation changed longitudinally and had differential impact in terms of publication, citations, or patent filings?

**Findings:**

In this cohort study of 8897 NIMH-funded R01, R21, and R03 projects investigated using a large language model, grants reflecting different RDoC domains differed substantially in their scientific impact in terms of publications, citations, and patent generation.

**Meaning:**

The findings suggest that large language models represent a promising approach to characterizing research proposals at scale, which may be useful in guiding resource allocation to maximize scientific return on investment.

## Teaser

This cohort study uses a large language model to examine changes in research funding from the National Institute of Mental Health (NIMH) for Research Domain Criteria domains and transdiagnostic investigation.

## Introduction

The Research Domain Criteria (RDoC) advanced by Insel and colleagues in 2009^1^ posited the utility of investigating psychiatric disorders in terms of a dimensional rather than categorical perspective. Identifying an initial 5 domains posited to be most closely linked to underlying biology, the RDoC framework emphasized the utility of cross-disorder investigation as well as the relevance of examining the continuity between health and psychopathology.

In a variety of contexts, the National Institute of Mental Health (NIMH) leadership has advocated for more widespread application of the RDoC framework,^2^ encouraging research proposals informed by this dimensional perspective. However, the extent to which this advocacy has been reflected in funding is unknown, as is whether this investment has yielded science with a differential impact. If the ultimate goal of NIMH investment is to diminish the burden of psychiatric disease,^3^ quantifying the effects of NIMH initiatives is critical. While funding decisions are premised on peer review, institutional leadership can shape the portfolio of an institution in a variety of ways and has been criticized for doing so in the past.^4^ This study therefore sought to investigate whether the emphasis by the largest funder of mental health research worldwide on the RDoC framework has shaped funding and impact over more than a decade.

## Methods

In this longitudinal cohort study, I queried the National Institutes of Health (NIH) RePORTER database between January 2003 and December 2023 using the ExPORTER application programming interface to retrieve all research proposals categorized as R01, R21, or R03 for which the NIMH was the primary funding entity. As this was bibliometric research not involving human participants, ethical review was not pursued. I followed the Strengthening the Reporting of Observational Studies in Epidemiology (STROBE) reporting guideline for cohort studies. The data included project titles and 30-line abstracts submitted by each study’s principal investigator for public dissemination at the time of grant application. Total direct-cost investment in each project was determined by summing over all project years. Administrative supplements (type 3, as described in the RePORTER data dictionary) were included in the parent grant. All dollar values were adjusted to 2023 values according to the multipliers in the Biomedical Research and Development Price Index^5^ based on prior evidence that failing to adjust for inflation could yield biased estimates of secular trends.^6^ For grants that were ongoing in 2024, I imputed total funding for the full grant period based on project start and end dates (eg, since only the first 3 years of a 5-year grant funded in 2021 would be included in RePORTER data, I assumed an additional 2 years based on mean funding level in the first 3 years).

To estimate RDoC domain coverage of a given project, I applied a method previously validated for narrative clinical notes in child, adolescent, and adult psychiatric populations^7^ that leverages the observation that the large language foundational model (ie, without any fine-tuning) includes training reflecting the individual RDoC domains such that the model can explain these domains. I used a Python script to access the chat.ai application programming interface to prompt a large language model (GPT4-turbo or turbo-2024-04-09 [OpenAI]) with temperature set at 0 and with individual titles and abstracts. The prompt was modified to categorize proposals as not addressing a given domain, addressing a given domain but not as a focus, or focusing on a given domain. I also asked whether a given proposal encompassed multiple diagnoses to determine whether it adopted a transdiagnostic perspective. The prompt was as follows:

You are a skilled psychiatrist scoring a research abstract in terms of how the aims reflect the 6 NIMH Research Domain Criteria (RDoC): negative valence systems, positive valence systems, cognitive systems, social processes, arousal and regulatory systems, and sensorimotor systems. Score 0 if concepts in a domain are not addressed, 0.5 if they are mentioned but not a focus, and 1 if they are a focus. A given abstract can have more than 1 focus. Do not score the processes or activities of the investigator, like meetings or training, just the content of the proposal. Do not infer involvement of a particular RDoC domain from the biological pathways discussed, just score explicit references to RDoC domains or symptoms or behaviors loading in that domain. Remember that substance use and addiction are reflected on the positive valence domain. Also score the extent (0 = not at all; 0.5 = somewhat; 1 = fully) to which aims integrate between multiple diagnoses.

As further validation, I blindly scored 150 randomly selected title-abstract combinations for each domain, with prevalence of each domain or concept reflecting that estimated in the full dataset (ie, the dataset was down-sampled to maintain the estimated prevalence for any given domain). Cohen κ values^8^ for agreement between the blind rating and the model’s assignment were 0.76 for negative, 0.79 for positive, 0.75 for cognitive, 0.83 for social, 0.75 for arousal, and 0.64 for sensorimotor domains and 0.66 for transdiagnostic proposals.

To quantify the impact of each project in terms of publications, I queried the list of PubMed identifiers (PMIDs) associated with each project in RePORTER. I then queried the European PubMed Central Application Programming Interface for each PMID, which allowed determination of the number of publications citing a given PMID each year. As such, I could quantify not only the number of articles resulting directly from a given project but also how impactful (in terms of citations) those articles would be. My primary metric for impact was a given grant’s h-index,^9^ estimated at 5 years from the index year of funding. That is, for each grant, I calculated the value *h* reflecting the number of articles each cited at least that many times. Because some lag in publication following study initiation is expected, with varying follow-up periods depending on date of funding initiation (essentially right-censoring these values), I conducted sensitivity analyses restricted to funding dates prior to January 2019 and excluding from analysis grants for which complete follow-up was not available. I also examined the h-index at a longer time horizon (10 years) and the total and annual numbers of citations. I determined the number of filed patents for each grant based on RePORTER data; given marked right skew in these values, I elected a priori to dichotomize the values to indicate presence or absence of at least 1 patent.

### Statistical Analysis

For descriptive purposes, I visualized the proportion of funded R01, R21, and R03 proposals over time, focusing on each RDoC domain and on transdiagnostic grants and plotting 3-year lagged rolling averages (ie, reflecting the mean of the current year and past 2 years). I used multiple linear regression to examine the association between individual RDoC domains and measures of scientific impact, including number of publications, h-index, and presence or absence of patents. (Sensitivity analyses also examined publications per year, total citations, and total citations per year.) All regression models were adjusted for index funding year, grant type (R01, R21, or R03), principal investigator structure (single or multiple), and total funding for that project. Analyses used R, version 4.3.2 (R Project for Statistical Computing),^10^ and 2-tailed *P* < .05 was used as the threshold for statistical significance.

## Results

Among 8897 R01, R03, and R21 projects initially funded between 2003 and 2023 (Table), accounting for $17.7 billion of investment in 2023 dollars, abstracts of 3141 (35.3%) reflected negative valence; 1344 (15.1%), positive valence; 2781 (31.3%), cognition; 1607 (18.1%), social; 343 (3.9%), arousal; and 571 (6.4%), sensorimotor domains. A total of 1793 (20.2%) incorporated a transdiagnostic perspective.

**Table.** Characteristics of NIMH-Funded R01, R03, and R21 Grants, 2003-2023

Feature	Grants (N = 8897)^a^
RDoC domains	
Negative	3141 (35.3)
Positive	1344 (15.1)
Cognitive	2781 (31.3)
Social	1607 (18.1)
Arousal	343 (3.9)
Sensorimotor	571 (6.4)
Transdiagnostic proposal	1793 (20.2)
Funding type	
R01	5703 (64.1)
R03	824 (9.3)
R21	2370 (26.6)
Total funding amount per $100 000, mean (SD)^b^	22.05 (19.64)
Direct funding amount per $100 000^b^	
Unavailable (before 2012)	3754 (42.2)
Mean (SD)	15.61 (13.96)
Multiple PIs	1654 (18.6)
Years funded, mean (SD)	3.60 (1.41)
Total publications, mean (SD)	10.74 (18.99)
Citations per year, mean (SD)	3.74 (4.87)
h-Index for grant, mean (SD)	5.27 (5.98)
≥1 Patent	229 (2.6)

Abbreviations: NIMH, National Institute of Mental Health; PI, principal investigator; RDoC, Research Domain Criteria.

^a^ Data are presented as number (percentage) of grants unless otherwise indicated.

^b^ Adjusted to 2023 dollars; total values were imputed if grants would have data for years beyond 2024 that were not yet available in the National Institutes of Health RePORTER database.

**Figure 1.**
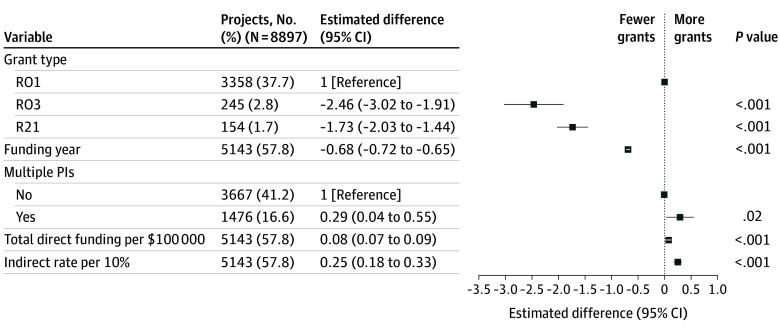
Rolling Average Proportion of National Institute of Mental Health Research Grants Addressing Each Research Domain Criteria Domain PI indicates principal investigator.

Figure 1 shows the proportion of grants reflecting a focus on each domain over time using a 3-year rolling average. Between 2003 and 2023, the greatest magnitude of increase was observed for the proportion of proposals that were categorized as transdiagnostic, with a mean increase of 0.011 (95% CI, 0.009-0.011) per year (ie, approximately 1.1% of total grants per year), followed by those with an RDoC of positive valence, with an increase of 0.005 (95% CI, 0.004-0.006) per year. Statistically significant but lesser increases were observed for negative valence (0.002; 95% CI, 0.006-0.004), cognition (0.003; 95% CI, 0.001-0.004), and social (0.003; 95% CI, 0.002-0.004) domains. No difference in the proportion of grants focused on the sensorimotor domain was observed (−0.002; 95% CI, −0.003 to 0.001). Repeating these analyses examining the proportion of funding rather than the proportion of the number of grants yielded similar results (eFigure 1 in Supplement 1).

**Figure 2.**
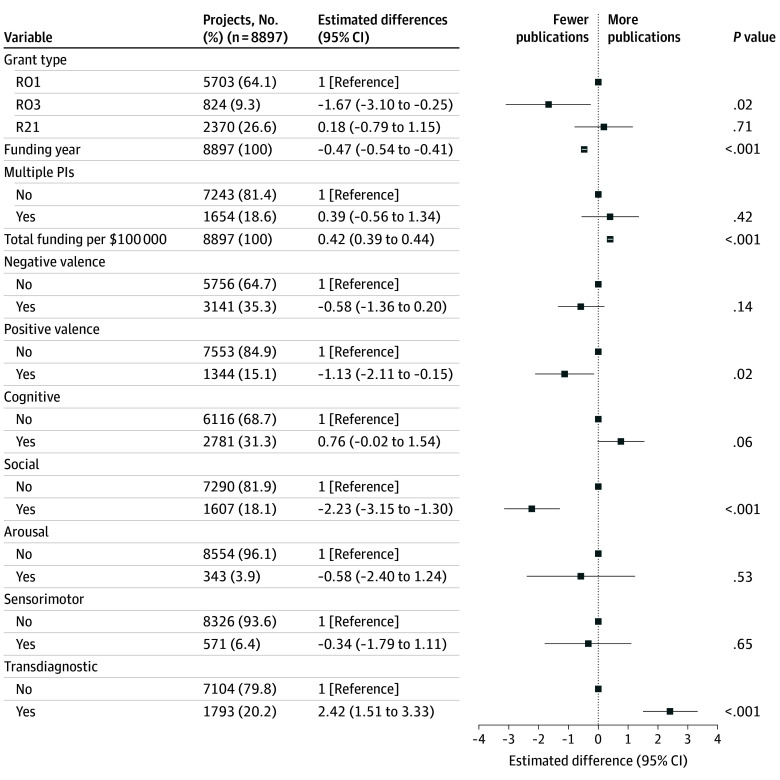
Linear Regression Model of Total Number of Publications Per Research Grant PI indicates principal investigator.

In multiple linear regression models, grants addressing the positive valence and social domains were associated with significantly fewer publications (difference, −1.13 [95% CI, −2.11 to −0.15] and −2.23 [95% CI, −3.15 to −1.30], respectively) (Figure 2), while transdiagnostic grants were associated with a significantly greater number of publications (difference, 2.42; 95% CI, 1.51-3.33). Grants addressing the social domain were associated with fewer publications on average (difference, −2.23; 95% CI, −3.15 to −1.30). Sensitivity analyses examining variable follow-up by restricting the analysis to proposals funded before 2019 (eFigure 2 in Supplement 1) or considering mean publications per year rather than all future publications (eFigure 3 in Supplement 1) yielded directionally similar results.

**Figure 3.**
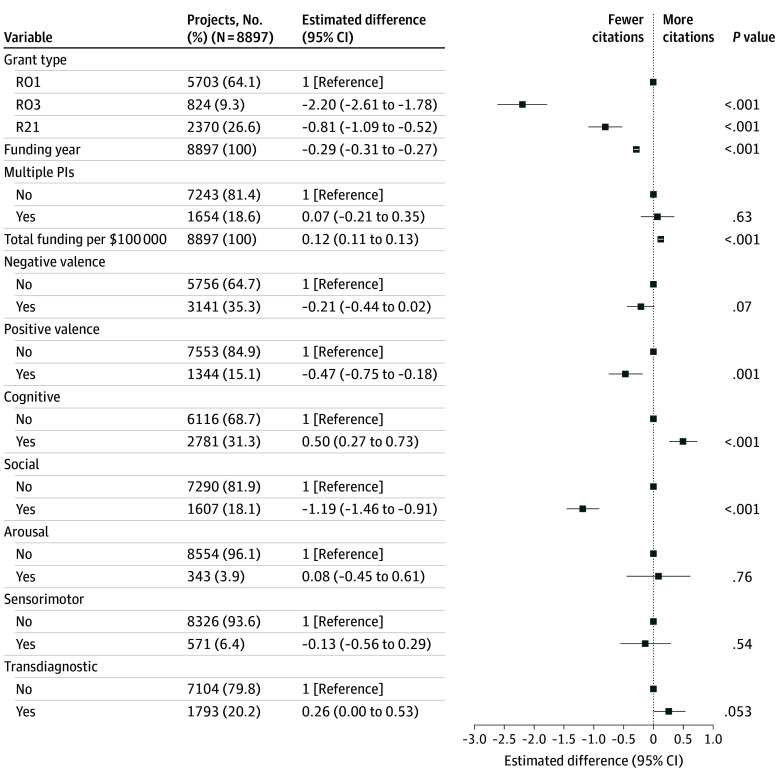
Linear Regression Model of Citation Impact Per Research Grant PI indicates principal investigator.

I next examined publication impact in terms of the 5-year h-index for each grant based on the number of citations of publications linked to a given proposal. As with the number of publications, the impact factors for positive valence (−0.47; 95% CI, −0.75 to −0.18) and social (−1.19; 95% CI, −1.46 to −0.91) domains were significantly lower than for proposals not focused on these domains (Figure 3). Cognition was associated with an increase in citation impact (0.50; 95% CI, 0.27-0.73). Restricting analyses to grants prior to 2019 (eFigure 4 in Supplement 1) yielded qualitatively similar results, as did analysis of the 10-year h-index (eFigure 5 in Supplement 1) or number of citations per year (eFigure 6 in Supplement 1).

**Figure 4.**
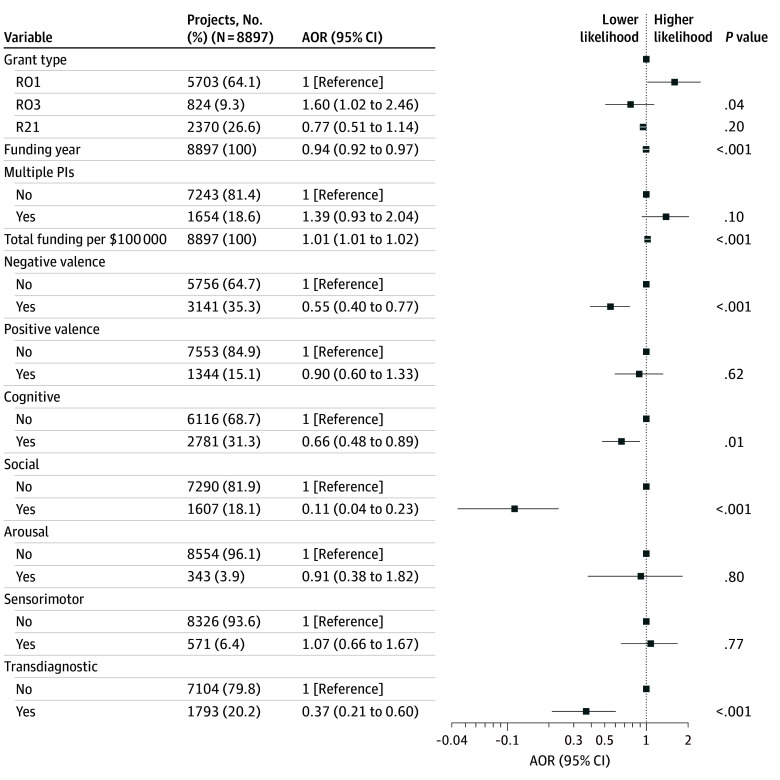
Logistic Regression Model of Likelihood of at Least 1 Patent Per Research Grant Models were adjusted for index funding year, grant type (R01, R21, or R03), principal investigator (PI) structure (single or multiple), and total funding for the project. AOR indicates adjusted odds ratio.

Finally, I examined the association between domain and likelihood of at least 1 patent linked to a grant. Figure 4 depicts a multiple logistic regression model of the association between individual domains and patent filing. Grants addressing the negative valence (adjusted odds ratio [AOR], 0.55; 95% CI, 0.40-0.77), cognition (AOR, 0.66; 95% CI, 0.48-0.89), and social (AOR, 0.11; 95% CI, 0.04-0.23) domains were significantly less likely to yield patents, as were those with a transdiagnostic approach (AOR, 0.37; 95% CI, 0.21-0.60). The magnitude of the effect size was greatest for the social domain, whereas grants not focused on this domain had odds of yielding a patent 9.1 times (1/0.11) greater than those addressing this domain. As with publications and citations, restricting the analysis to grants prior to 2019 yielded similar results (eFigure 7 in Supplement 1).

## Discussion

This study of R01, R03, and R21 grants funded by the NIMH between 2003 and 2023 identified an increased proportion of funded grant proposals reflecting the positive valence, negative valence, cognition, and social domains. An increasing proportion also reflected a transdiagnostic perspective. In aggregate, these shifts suggest that programmatic emphasis on RDoC concepts over the past decade has had the intended impact, eliciting more RDoC-focused fundable research proposals.

If such grants have increased as a proportion of overall NIMH R grant funding, have they been associated with a corresponding increase in scientific productivity? A key caveat in addressing this question is that such metrics focus on a narrow and specific definition of productivity, namely publications. Examining patents extends such analyses by adding a complementary measure of output, since only a small proportion of discoveries results in such intellectual property and many important contributions to science will not yield a patent at all. Still, these metrics do not capture the potential for broader influences of research informed by RDoC—for example, in facilitating clinical implementation of dimensional measures or changes in mental health policy to adopt a transdiagnostic perspective.

Nonetheless, I identified a notable decrease in publications associated with grants in particular RDoC domains, including the positive valence and social domains. The pattern for patents was somewhat different; social domains were again associated with reduction along with the negative valence and cognitive domains. One possible explanation for these outcomes is that they could reflect underlying patterns of citation for the diagnoses that load on these domains—for example, if topics like major depressive disorder (predominantly negative valence) are simply more widely cited than less-studied areas (eg, disorders of arousal). On the other hand, grants addressing a transdiagnostic perspective were associated with a greater number of publications but fewer patents. While speculative, this may reflect enthusiasm for new perspectives in psychiatry^11^ along with challenges in conducting such studies in a manner that yields patentable biotechnology.^12^

Few prior studies have focused quantitatively on NIMH investments. One prior report examined investment by disorder using an aggregated NIH database to calculate a decrease in support for schizophrenia and bipolar disorder between 2016 and 2021.^13^ Another report, the only one I was able to identify that used primary grant–level NIMH data, found that investment in child psychiatry investigation decreased by more than 40% between 2005 and 2015,^14^ though much of this change reflected inflation effects diminishing NIH purchasing power overall.^6^ The paucity of such studies is particularly surprising given the magnitude of NIH funding and the need to understand the return on investment, however defined. In capturing such trends, it would be important to distinguish overt shifts in NIMH priorities^3^ from advances in technology that open new areas for investigation (eg, genomics, artificial intelligence) and changes in public health concerns (eg, opioids, COVID-19).

### Limitations

This study has multiple limitations. The use of a large language model allowed a systematic evaluation of nearly 10 000 research abstracts but may still have introduced misclassification errors, as agreement with a blinded rater was substantial^8^ but far from perfect. As such, these results represent inexact estimates by definition. Misclassification would likely add noise and make associations more difficult to detect or would lead to underestimation of the magnitude of associations. Furthermore, while I elected to focus on the major extramural grant mechanisms that remain consistent over time, I omitted other, more specialized mechanisms (eg, UG3, UH3), center grants (U01), or certain training grants (eg, T32) that may be used in requests for applications seeking to target specific areas. Examining these narrower mechanisms would be a valuable next step in understanding shifts in funding priorities. Finally, although I used multiple sensitivity analyses to demonstrate the robustness of the effects, I cannot exclude the possibility that secular trends could contribute to the results I observed, since not all outcomes are fully observed. As there is likely no optimal approach for all of these contingencies, I elected simply to consider alternative strategies that illustrate the robustness of the associations I identified.

This study also could not examine the extent to which RDoC-related studies may be prioritized for extramural funding, as only data on funded studies are publicly available. I thus could not answer the complementary question about whether grant applications, regardless of success, have shifted over time. Peer reviewers are not necessarily aligned with NIMH leadership or with program staff in their priorities, such that particular types of grants (eg, those addressing particular RDoC domains or adopting a transdiagnostic perspective) may have differential success in review. Nonetheless, regardless of the extent to which the institute shapes funding decisions, understanding the way in which priorities may shift scientific productivity may be important in planning future initiatives.

## Conclusions

This study of NIMH funding between 2003 and 2023 found an expansion of funded proposals incorporating a transdiagnostic perspective and capturing specific RDoC domains. The results indicated that grants focusing on particular domains were associated with a diminution of scientific output. More broadly, the findings suggest the utility of an approach to applying large language models to facilitate quantification of shifts in NIMH funding priorities.

## Notes

Supplement 1. **eFigure 1.** Proportion of Total Research Funding Addressing Each RDoC Domain **eFigure 2.** Linear Regression Model of Number of Publications, Restricted to Grants Funded Prior to 2019 **eFigure 3.** Linear Regression Model of Number of Publications per Year **eFigure 4.** Linear Regression Model of 5-Year h-Index, Limited to Grants Funded Prior to 2019 **eFigure 5.** Linear Regression Model of 10-Year h-Index **eFigure 6.** Linear Regression Model of Number of Citations Per Year **eFigure 7.** Logistic Regression Model of Likelihood of at Least 1 Patent, Restricted to Grants Funded Prior to 2019

Supplement 2.
Data Sharing Statement
