# Supplementary material for: Research Domain Criteria in NIMH Grants Characterized Using Large Language Models
Source: JAMA Netw Open. 2025 Feb 12;8(2):e2459371. doi: 10.1001/jamanetworkopen.2024.59371 (PMC11822550; doi:10.1001/jamanetworkopen.2024.59371)
Supplement: Supplement 1. — eFigure 1. Proportion of Total Research Funding Addressing Each RDoC Domain eFigure 2. Linear Regression Model of Number of Publications, Restricted to Grants Funded Prior to 2019 eFigure 3. Linear Regression Model of Number of Publications per Year eFigure 4. Linear Regression Model of 5-Year h-Index, Limited to Grants Funded Prior to 2019 eFigure 5. Linear Regression Model of 10-Year h-Index eFigure 6. Linear Regression Model of Number of Citations Per Year eFigure 7. Logistic Regression Model of Likelihood of at Least 1 Patent, Restricted to Grants Funded Prior to 2019 [file jamanetwopen-e2459371-s001.pdf]

Supplementary Online Content

Perlis RH. Research Domain Criteria in NIMH grants characterized using large language models. *JAMA Netw Open*. 2025;8(2):e2459371. doi:10.1001/jamanetworkopen.2024.59371

eFigure 1. Proportion of Total Research Funding Addressing Each RDoC Domain
eFigure 2. Linear Regression Model of Number of Publications, Restricted to Grants Funded Prior to 2019
eFigure 3. Linear Regression Model of Number of Publications per Year
eFigure 4. Linear Regression Model of 5-Year h-Index, Limited to Grants Funded Prior to 2019
eFigure 5. Linear Regression Model of 10-Year h-Index
eFigure 6. Linear Regression Model of Number of Citations Per Year
eFigure 7. Logistic Regression Model of Likelihood of at Least 1 Patent, Restricted to Grants Funded Prior to 2019

This supplementary material has been provided by the authors to give readers additional information about their work.

© 2025 Perlis RH et al. *JAMA Network Open*.

eFigure 1. Proportion of total research funding addressing each RDoC Domain

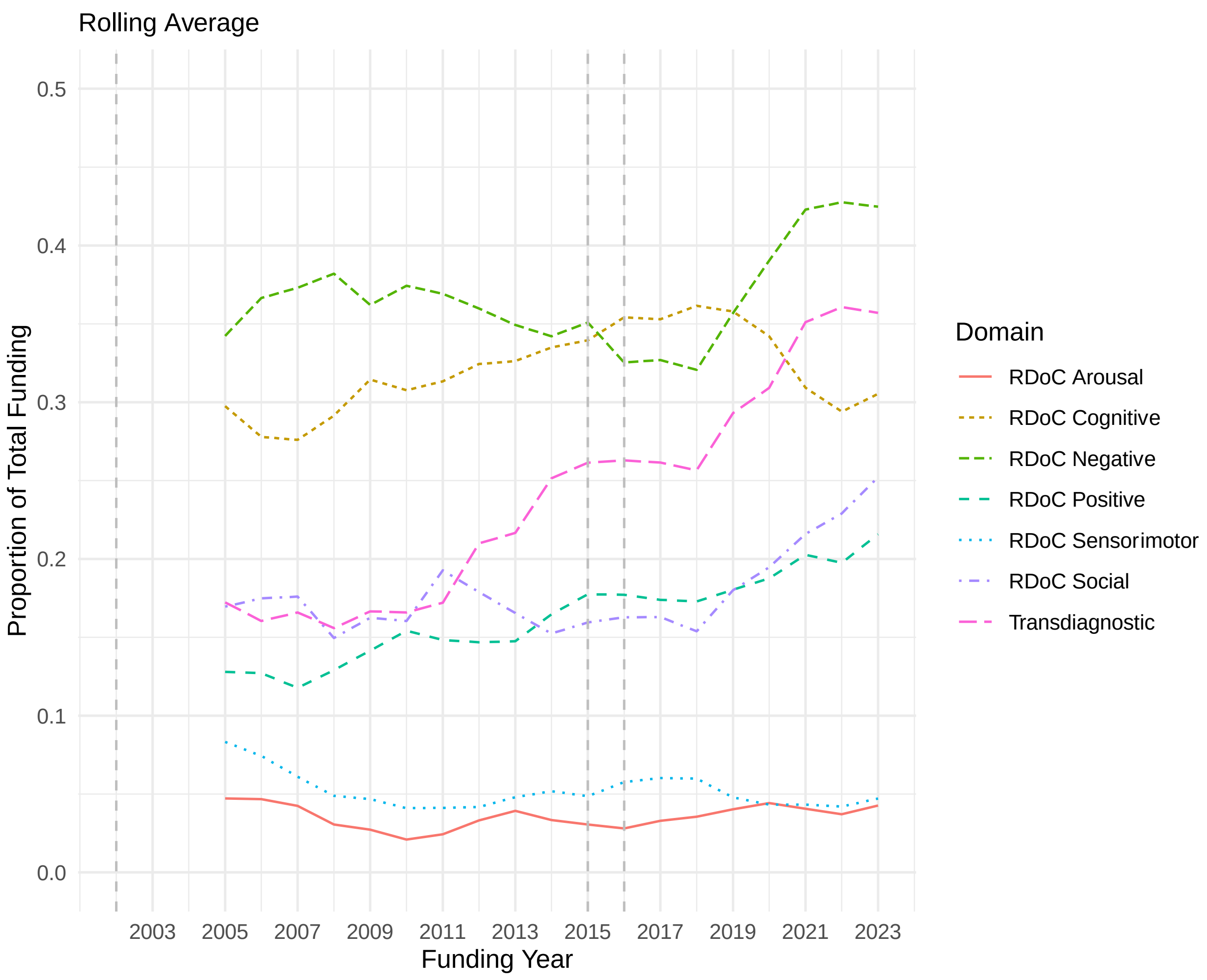

© 2025 Perlis RH et al. *JAMA Network Open*.

eFigure 2. Linear regression model of number of publications, restricted to grants funded prior to 2019

Variable		N	Estimate	p
**Grant Type**	R01	4146	Reference	
	R03	750	−2.41 (−4.15, −0.68)	0.006
	R21	1740	−1.38 (−2.69, −0.08)	0.038
**Funding Year**		6636	−0.23 (−0.34, −0.12)	<0.001
**Multi−PI**	No	5756	Reference	
	Yes	880	1.02 (−0.39, 2.44)	0.157
**Total Funding (per $100k)**		6636	0.42 (0.39, 0.45)	<0.001
**Negative**	No	4384	Reference	
	Yes	2252	−0.34 (−1.36, 0.68)	0.511
**Positive**	No	5723	Reference	
	Yes	913	−1.20 (−2.54, 0.13)	0.077
**Cognitive**	No	4594	Reference	
	Yes	2042	0.90 (−0.13, 1.92)	0.086
**Social**	No	5528	Reference	
	Yes	1108	−2.39 (−3.64, −1.14)	<0.001
**Arousal**	No	6392	Reference	
	Yes	244	−0.51 (−2.95, 1.93)	0.681
**Sensorimotor**	No	6182	Reference	
	Yes	454	−0.26 (−2.11, 1.59)	0.783
**Transdiagnostic**	No	5518	Reference	
	Yes	1118	3.27 (2.01, 4.53)	<0.001

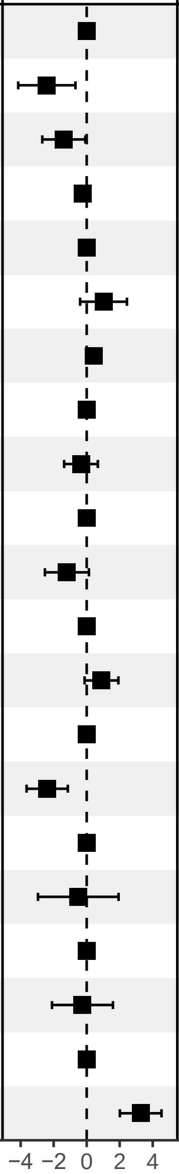

© 2025 Perlis RH et al. *JAMA Network Open*.

eFigure 3. Linear regression model of number of publications per year

Variable		N	Estimate	p
**Grant Type**	R01	5703	Reference	
	R03	824	−0.20 (−0.33, −0.07)	0.002
	R21	2370	−0.20 (−0.28, −0.11)	<0.001
**Funding Year**		8897	0.03 (0.02, 0.03)	<0.001
**Multi−PI**	No	7243	Reference	
	Yes	1654	0.15 (0.06, 0.23)	<0.001
**Total Funding (per $100k)**		8897	0.03 (0.03, 0.03)	<0.001
**Negative**	No	5756	Reference	
	Yes	3141	−0.08 (−0.14, −0.01)	0.032
**Positive**	No	7553	Reference	
	Yes	1344	−0.16 (−0.25, −0.07)	<0.001
**Cognitive**	No	6116	Reference	
	Yes	2781	0.02 (−0.05, 0.09)	0.520
**Social**	No	7290	Reference	
	Yes	1607	−0.27 (−0.35, −0.19)	<0.001
**Arousal**	No	8554	Reference	
	Yes	343	−0.11 (−0.27, 0.05)	0.188
**Sensorimotor**	No	8326	Reference	
	Yes	571	−0.03 (−0.16, 0.10)	0.647
**Transdiagnostic**	No	7104	Reference	
	Yes	1793	0.29 (0.21, 0.38)	<0.001

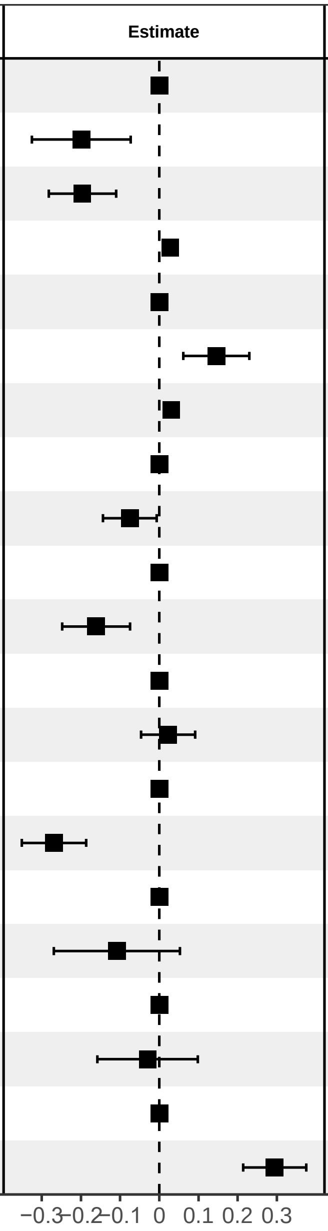

© 2025 Perlis RH et al. *JAMA Network Open*.

eFigure 4. Linear regression model of 5-year h-index, limited to grants funded prior to 2019

Variable		N	Estimate		p
**Grant Type**	R01	4146		Reference	
	R03	750		−2.80 (−3.30, −2.30)	<0.001
	R21	1740		−1.80 (−2.18, −1.43)	<0.001
**Funding Year**		6636		−0.13 (−0.16, −0.10)	<0.001
**Multi−PI**	No	5756		Reference	
	Yes	880		0.26 (−0.15, 0.66)	0.21
**Total Funding (per $100k)**		6636		0.11 (0.11, 0.12)	<0.001
**Negative**	No	4384		Reference	
	Yes	2252		−0.09 (−0.38, 0.20)	0.54
**Positive**	No	5723		Reference	
	Yes	913		−0.49 (−0.87, −0.10)	0.01
**Cognitive**	No	4594		Reference	
	Yes	2042		0.59 (0.30, 0.89)	<0.001
**Social**	No	5528		Reference	
	Yes	1108		−1.35 (−1.71, −0.99)	<0.001
**Arousal**	No	6392		Reference	
	Yes	244		0.29 (−0.41, 0.99)	0.42
**Sensorimotor**	No	6182		Reference	
	Yes	454		−0.10 (−0.63, 0.43)	0.70
**Transdiagnostic**	No	5518		Reference	
	Yes	1118		0.43 (0.07, 0.79)	0.02

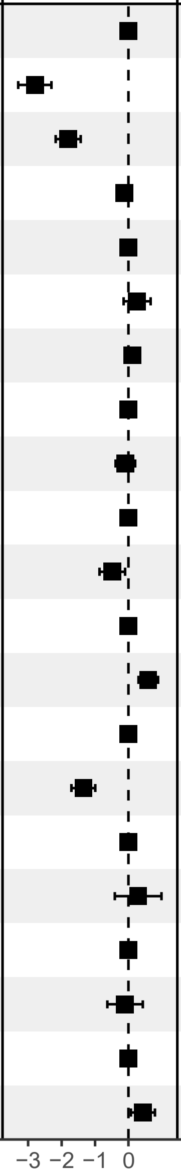

© 2025 Perlis RH et al. *JAMA Network Open*.

eFigure 5. Linear regression model of 10-year h-index

Variable		N	Estimate		p
**Grant Type**	R01	5703		Reference	
	R03	824		−2.51 (−3.00, −2.03)	<0.001
	R21	2370		−0.88 (−1.21, −0.55)	<0.001
**Funding Year**		8897		−0.36 (−0.39, −0.34)	<0.001
**Multi−PI**	No	7243		Reference	
	Yes	1654		0.02 (−0.30, 0.35)	0.890
**Total Funding (per $100k)**		8897		0.14 (0.13, 0.15)	<0.001
**Negative**	No	5756		Reference	
	Yes	3141		−0.19 (−0.45, 0.07)	0.160
**Positive**	No	7553		Reference	
	Yes	1344		−0.45 (−0.78, −0.11)	0.009
**Cognitive**	No	6116		Reference	
	Yes	2781		0.63 (0.37, 0.90)	<0.001
**Social**	No	7290		Reference	
	Yes	1607		−1.22 (−1.53, −0.90)	<0.001
**Arousal**	No	8554		Reference	
	Yes	343		0.13 (−0.49, 0.75)	0.675
**Sensorimotor**	No	8326		Reference	
	Yes	571		−0.14 (−0.63, 0.35)	0.578
**Transdiagnostic**	No	7104		Reference	
	Yes	1793		0.37 (0.06, 0.68)	0.020

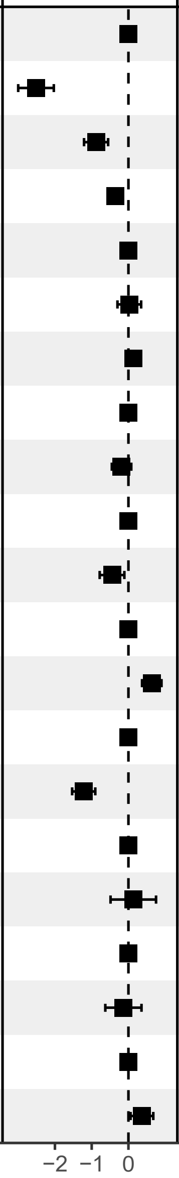

© 2025 Perlis RH et al. *JAMA Network Open*.

eFigure 6. Linear regression model of number of citations per year

Variable		N	Estimate	p
**Grant Type**	R01	5703	Reference	
	R03	824	−4.02 (−11.83, 3.79)	0.313
	R21	2370	2.38 (−2.94, 7.70)	0.380
**Funding Year**		8897	−1.24 (−1.59, −0.89)	<0.001
**Multi−PI**	No	7243	Reference	
	Yes	1654	2.19 (−3.04, 7.41)	0.412
**Total Funding (per $100k)**		8897	1.64 (1.51, 1.77)	<0.001
**Negative**	No	5756	Reference	
	Yes	3141	−10.59 (−14.84, −6.33)	<0.001
**Positive**	No	7553	Reference	
	Yes	1344	−12.91 (−18.29, −7.53)	<0.001
**Cognitive**	No	6116	Reference	
	Yes	2781	−5.10 (−9.38, −0.82)	0.020
**Social**	No	7290	Reference	
	Yes	1607	−18.08 (−23.17, −12.98)	<0.001
**Arousal**	No	8554	Reference	
	Yes	343	−0.04 (−10.02, 9.95)	0.994
**Sensorimotor**	No	8326	Reference	
	Yes	571	−9.66 (−17.61, −1.70)	0.017
**Transdiagnostic**	No	7104	Reference	
	Yes	1793	6.73 (1.74, 11.72)	0.008

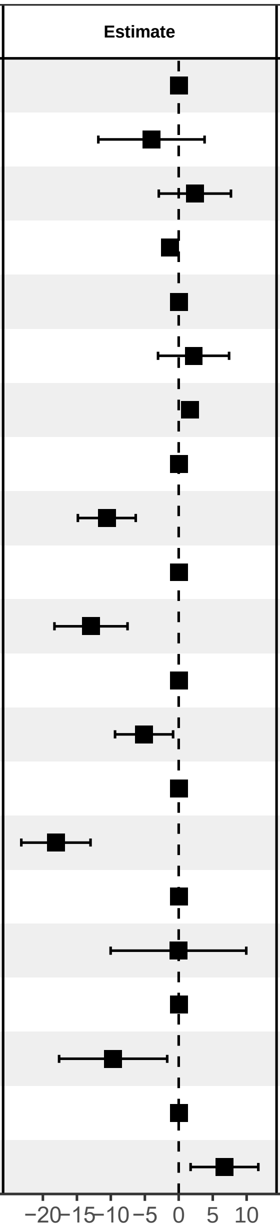

© 2025 Perlis RH et al. *JAMA Network Open*.

eFigure 7. Logistic regression model of likelihood of at least 1 patent, restricted to grants funded prior to 2019

Variable		N	Odds ratio		p
**Grant Type**	R01	4146		Reference	
	R03	750		1.54 (0.98, 2.38)	0.054
	R21	1740		0.72 (0.48, 1.07)	0.117
**Funding Year**		6636		1.01 (0.98, 1.04)	0.532
**Multi−PI**	No	5756		Reference	
	Yes	880		1.29 (0.87, 1.89)	0.195
**Total Funding (per $100k)**		6636		1.01 (1.01, 1.02)	<0.001
**Negative**	No	4384		Reference	
	Yes	2252		0.58 (0.41, 0.80)	0.001
**Positive**	No	5723		Reference	
	Yes	913		0.92 (0.61, 1.35)	0.686
**Cognitive**	No	4594		Reference	
	Yes	2042		0.65 (0.47, 0.88)	0.006
**Social**	No	5528		Reference	
	Yes	1108		0.12 (0.05, 0.25)	<0.001
**Arousal**	No	6392		Reference	
	Yes	244		0.96 (0.40, 1.93)	0.916
**Sensorimotor**	No	6182		Reference	
	Yes	454		1.09 (0.67, 1.70)	0.713
**Transdiagnostic**	No	5518		Reference	
	Yes	1118		0.38 (0.22, 0.63)	<0.001

Odds ratio axis: 0.05 0.1 0.2 0.5 1 2

© 2025 Perlis RH et al. *JAMA Network Open*.
