# Supplementary material for: Research Domain Criteria in NIMH Grants Characterized Using Large Language Models
Source: JAMA Netw Open. 2025 Feb 12;8(2):e2459371. doi: 10.1001/jamanetworkopen.2024.59371 (PMC11822550; doi:10.1001/jamanetworkopen.2024.59371)
Supplement: Supplement 2. — Data Sharing Statement [file jamanetwopen-e2459371-s002.pdf]

Data Sharing Statement

Perlis. Research Domain Criteria in NIMH Grants Characterized Using Large Language Models. *JAMA Netw Open*. Published February 12, 2025.
doi:10.1001/jamanetworkopen.2024.59371

Data
Data available: No

Additional Information
Explanation for why data not available: All data used for this analysis is already publicly available, which is indicated in the data sharing statement in the manuscript.
